# Vehicle Detection in Aerial Images Using a Fast Oriented Region Search and the Vector of Locally Aggregated Descriptors

**DOI:** 10.3390/s19153294

**Published:** 2019-07-26

**Authors:** Chongyang Liu, Yalin Ding, Ming Zhu, Jihong Xiu, Mengyang Li, Qihui Li

**Affiliations:** 1 Key Laboratory of Airborne Optical Imaging and Measurement, Changchun Institute of Optics, Fine Mechanics and Physics, Chinese Academy of Sciences, Changchun 130033, China; 2 University of Chinese Academy of Sciences, Beijing 100049, China

**Keywords:** vehicle detection, aerial images, object proposals, vector of locally aggregated descriptors (VLAD)

## Abstract

Vehicle detection in aerial images plays a significant role in civil and military applications and it faces many challenges including the overhead-view perspective, the highly complex background, and the variants of vehicles. This paper presents a robust vehicle detection scheme to overcome these issues. In the detection stage, we propose a novel algorithm to generate oriented proposals that could enclose the vehicle objects properly as rotated rectangles with orientations. To discriminate the object and background in the proposals, we propose a modified vector of locally aggregated descriptors (VLAD) image representation model with a recently proposed image feature, i.e., local steering kernel (LSK) feature. By applying non-maximum suppression (NMS) after classification, we show that each vehicle object is detected with a single-oriented bounding box. Experiments are conducted on aerial images to compare the proposed method with state-of-art methods and evaluate the impact of the components in the model. The results have proven the robustness of the proposed method under various circumstances and the superior performance over other existing vehicle detection approaches.

## 1. Introduction

With the improvement of airborne camera and remote sensing system, high-resolution aerial images captured by un-manned airborne vehicles (UAVs) or satellites are trending to be more common recently. These aerial images provide lots of data for researchers, therefore in this paper, we focus on vehicle detection in overhead imagery. This task has been a standing topic for several decades owing to the demands of gathering crucial information of targets in many applications, such as traffic surveillance, road assistance, urban planning, optical mapping, and military reconnaissance [1,2]. However, vehicle detection in aerial images still faces many challenges.

**Figure 1:**
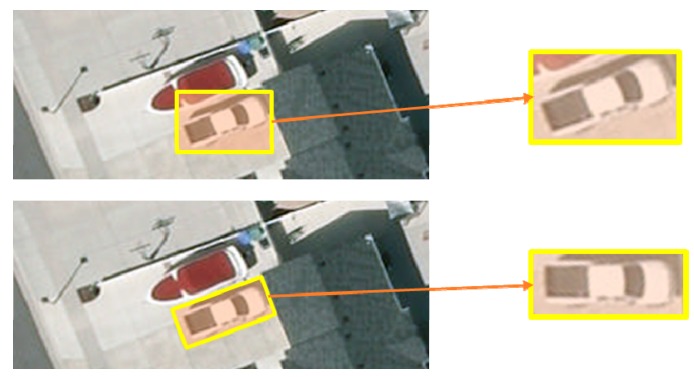
Illustration of horizontal RoI (top) and oriented RoI (bottom) in an aerial image.

First, objects in aerial images captured in overhead view could reflect much less information than in generic images captured with horizontal view. One of the main factors is the remote distance, while different resolution images display different information, the details are considerably limited even at very high resolution. For images captured by satellites or high-altitude UAVs, the resolution with the ground sampling distance (GSD) is usually lower than 1 m. For instance, the average size of a car is typically 48 × 16 pixels in an aerial image dataset with a resolution of 0.1 m [2]. For images captured using low-altitude UAVs, an astonishing resolution can be achieved with the GSD at the centimeter-level. For instance, if the length and width are around 4.5 and 1.8 m, the size of the car is 180 × 80 pixels in an aerial image with a sensor resolution of 2 cm [3]. Satellite and UAV images have very different properties due to the difference in GSD [1]. Thus, many satellite-based approaches are not compatible with UAV-based approaches, and vice versa. Second, aerial images are filled with many objects similar to vehicle targets, such as electrical units, roofs of rectangular buildings, trash bins, and air conditioning units. These objects are more likely to cause false positive errors, which makes it more difficult to discriminate targets in the background. Third, vehicles are placed in arbitrary orientations. In the past, most vehicle detection or algorithms calculate the orientation independently or aim at counting the vehicles and localizing the position using horizontal bounding boxes as region of interests (RoIs) without orientation. However, it is precisely because of the diversity and randomness of the orientation that it is very different from generic object detection. As shown in Figure 1, the horizontal RoI (HRoI) typically encloses a vehicle target as most detection methods do, yet we can observe that some of the background and a part of nearby vehicle object are contained in the HRoI. Objects being tightly packed is a common phenomenon in aerial images, where the interference is detected during feature extracting under this circumstance, which affects the accuracy of the detection results. On the contrary, using oriented bounding boxes to represent RoIs not only eliminates the possibility of containing uninterested parts but also reduces the diversity owing to all the oriented RoIs (ORoIs) lying in the same orientation after rotating to the baseline axis.

**Figure 2:**
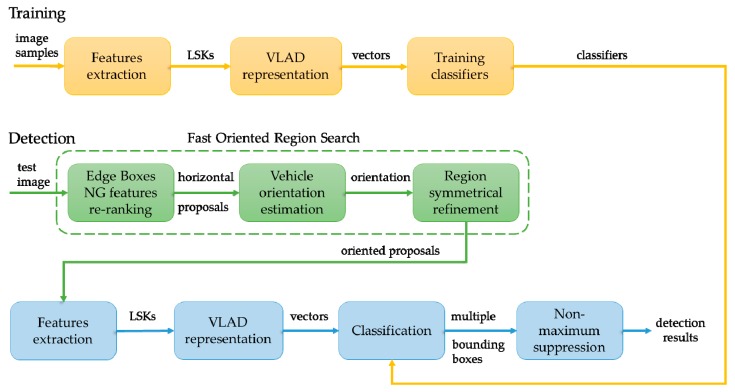
Proposed vehicle detection framework.

In this paper, we propose a robust vehicle detection method in aerial images. This approach can detect objects with ORoIs and recognize them, even when the background is highly complex in various aerial images, including satellite and UAV images. Refer to Figure 2, which illustrates the framework of our method. In order to detect the positions, sizes, and orientations of objects, we propose an algorithm namely Fast Oriented Region Search. As we demonstrate and validate in the following sections, a local steering kernel (LSK) is a suitable feature to apply in this task. Instead of representing an object by a single feature vector, we regard a vehicle as an aggregation composed of a variety of visual words. These visual words are associated with different entities (e.g., roofs, wheels, shells) of a category of vehicle. This idea is inspired by the vector of locally aggregated descriptors (VLAD), which already exhibits remarkable advantages in image retrieval and scene recognition applications. Therefore, we adapted the VLAD theory into vehicle detection by evaluating its properties in representing images. In the experimental part, we test and evaluate our method, and compare it with other state-of-art methods on two publicly-available datasets, as well as other aerial images. Moreover, we also conduct experiments to compare our proposed generating algorithm with other objectness approaches.

In summary, our contributions are the following:

- We propose an algorithm to generating ORoIs called a Fast Oriented Region Search, which is composed of Edge Boxes NG features re-ranking, vehicle orientation estimation, and region symmetrical refinement. This approach is efficient and accurate, which is significant for the subsequent steps.
- By introducing a dense feature extraction approach based on LSK into a VLAD model, we achieve a better representation of the vehicle objects in the image owing to LSK being designed to be invariant to variations. The properties of LSK guarantees the robustness and stableness of our method.
- In view of a large variety of vehicle categories and the difficulty of imbalanced samples, we optimize the training phase. The classification results were improved by applying a modified directed-acyclic-graph support vector machine (DAG SVM) approach, which is trained with negative samples and multiple categories of vehicle samples.

## 2. Related Work

Vehicle detection in aerial images is quite different from generic object detection owing to its characteristic of objects and background. In the literature, early studies focus more on the low-resolution satellite images, which is restricted by the technology of that time. Some features that aim to represent vehicle profiles like boundaries of the car body and windshield, shadows, and intensity of illumination, etc., are often used due to the limited information and details. Zhao and Nevatia [3] proposed a method of integrating these car-profile features into a Bayesian network to detect cars. In Hinz [4], the candidates of vehicle targets are carried out by a top-down algorithm. Hinz built a model based on local features, global features, and a vehicle queue. 3D-wireframe representation including shadow areas is introduced for describing the vehicle target in geometric and radiometric level. Choi and Yang [5] introduced a method to extract initial vehicle candidates based on a mean-shift algorithm with well-proportioned car structure features in blob-like shapes. The log-polar shape descriptor is used for the connection of blob candidates and vehicles. In X. Niu [6], a geometric deformable model is used to design a seed point propagation framework, which is based on an edge map extracted by Canny edge detector. In Kozempel and Reulke [7], a fast detection method is provided for traffic objects, and the car model is also based on an edge map but represented by four-shaped edge filters. This allows it to achieve a better performance in detecting cars of different sizes and orientation. In addition, morphology has a good effect in dealing with the detection of small and weak targets [8]. Z. Zheng [9] proposed a robust and automatic vehicle detection method for highway aerial images based on morphology, and H. Zheng [10] used a morphological neural network to extract vehicle targets from high-resolution satellite images.

Since those low-level features (i.e., boundaries, edges, intensities, shadows) are inadequate for representing a vehicle object in an aerial image with a complex background, early studies of vehicle detection face many challenges, such as the similarities of rectilinear and car structures, and further classification of vehicles (i.e., cars, trucks, boats, and aircrafts). With the development of image descriptors of features in computer vision, advanced features achieve promising performance in general object detection tasks, which leads researchers to adapt and fit in mature features in a vehicle detection framework.

In Breckon [11], a two-stage approach to achieve autonomous vehicle detection is introduced. The authors trained multiple cascaded Haar-like classifiers to evaluate vehicles at different scales and positions. Then, they complete the verification stage based on the UAV’s altitude driven vehicle size constraints, a so-called a fuzzy logic style. S. Tuermer et al. [12] proposed an automatic vehicle detection in high-resolution aerial images captured by a 3K camera system. To limit the search space for the detector, the authors smooth the RGB color image with a Gaussian filter and calculate the Euclidean distance of each two pixels in the pre-processing image. Then they generate a reliable detector with 2D and 3D histograms of oriented gradient (HOG) features on two consecutive images, and perform the classification by using AdaBoost. In Sahli [13], the scale-invariant feature transform (SIFT) is used to extract key points in the low-resolution aerial image. Then, the SVM is used to classify the SIFT descriptor of potential candidates. A method to obtain the principal orientation of the car is also introduced by computing the gradients at each pixel to build a histogram of orientations of 24 bins. In Leitloff [14], a Haar-like feature-based adaptive boosting classifier was constructed using prior knowledge of typical vehicle characteristics. In Grabner [1], an automatic car detection framework is presented based on an on-line boosting algorithm. The classifier was trained by multiple image feature descriptors, including Haar-like, HOG, and local binary patterns (LBP) features. In some studies, additional information was considered to make the vehicle detection approaches more accurate, such as constraining searching area on the road by using geographic information system (GIS) data [15,16], localizing the position of vehicle targets by measuring the movement speed [17], and evaluating the correlation of cars in consecutive frames [2]. In this paper, we focus on vehicle detection in a single aerial image.

With the development of UAV technology (i.e., airborne camera, platform, and remote sensing system), different resolutions of UAV images have received great attention from geoscience and remote sensing researchers [18]. Moranduzzo and Melgani have suggested several ways to achieve the aim of vehicle detection in UAV images. At the beginning, their work [19] was based on key-point SIFT features with an SVM classifier, where the descriptors were simply divided into two groups: cars or non-cars. Afterwards, their refined work [20] further improved the performance of their previous work. By taking advantage of a priori knowledge of asphalted areas, the screening operation not only accelerated the screening time, but also enhanced the performance. To ensure the principle of each car object only having one key-point, a key-point merging algorithm was presented to delete the duplicate key-points in car candidates. Unlike image matching or image retrieval, it was observed that dense features can perform better than sparse features in object detection or scene recognition. Moranduzzo and Melgani finally improved their work by using HOG features and a 36-bin orientation estimation [21].

For the purpose of enhancing the representation power, aggregating algorithms that could combine features like bag-of-visual-words (BoW), Fisher Vector, VLAD, etc., were introduced to encode local image descriptors. These encoding methods are highly mature in many fields, but are rarely used in vehicle detection. In Moranduzzo [21], a similar idea was mentioned: a vehicle object could be viewed as composed of a small vocabulary of visual words. Then, a histogram with corresponding scores was produced by measuring the similarity between potential patches and visual words in the vocabulary, and used to train the SVM classifier. Unlike the BoW model, features related to local components of a car are not used in their work.

As for estimating the orientations of vehicles, existing methods usually train the classifiers several times in multiple orientations or repeat the screening procedure in all the possible directions. In Breckon [11], a training set was divided into groups based on four angular offsets—0°, 45°, 90°, and 135°—and the subsets were used for training the multiple classifiers. In Grabner [1], the localizing algorithm in the screening phase was rotated with an angle interval of 10°. In Moranduzzo [21], the orientation was confirmed by searching for the nearest value in 36 possible directions. Recently in Luo [22], each test image patch was rotated after their main direction was estimated. This makes the whole pipeline avoid redundant locating or training procedures. Edges and lines were detected using Canny and Hough transforms, and then clustered into groups according to their angles. The angle that had the most lines was taken as the main direction.

## 3. Proposed Method

### 3.1. Overview of the Proposed Method

Like most detection frameworks based on machine learning, our method mainly contains two stages: training and detection. During the training stage, the purpose is to make full use of the samples to train the classifiers. As for the detection stage, our method consists of two modules: generating oriented proposals that almost cover all the targets, and classifying proposals according to the trained classifier models.

(1) For training, we first generated positive and negative samples. Second, the LSK features were densely computed because dense sampling strategies are capable of producing more information than key-point-based strategies during the feature-extraction phase, especially for detecting small targets like vehicles in aerial images, where the patches do not have sufficient key-points to be extracted. The reason for using LSK features is that LSK can better capture the characteristics of vehicles under different conditions, even when the target is influenced by illumination, noise, and blur. Third, a VLAD representation was constructed by using a K-means algorithm to build a codebook of visual words. By measuring and accumulating the distance between codewords and descriptors after the principal component analysis (PCA) process, VLAD encoded vectors to characterize the correlation of a descriptor and the visual content. After all the samples were encoded, we could obtain classifiers trained with VLAD vectors.
(2) A Fast Oriented Region Search was first used to generate horizontal bounding box candidates quickly with as few aerial images as possible by applying Edge Boxes NG features re-ranking (EBNR), then calculated the orientations by applying a vehicle orientation estimation. If we simply rotated horizontal bounding boxes to their main directions, the rotated bounding boxes may not be accurately enclosing the objects because the vehicle targets may lie in the rectangular diagonal of boxes. To address this problem, we introduced a fast algorithm to get a point-set of a vehicle targets that could represent its contours. By rotating the points in the set and the corresponding superpixel box, we were able to achieve box refinement. A highlight of this approach is that it only needs to get the superpixel segment graph one time and compute between the bounding boxes. Therefore, this method significantly saves computational time to get oriented proposals efficiently.
(3) Discriminating the ORoIs is the primary mission in this classifying stage. We computed the LSK features and encoded them based on the codebook we built in the training stage. Then, we use the classifiers to recognize the objects in the ORoIs. The classification mechanism was based on ranking scores where one object was very likely surrounded by many oriented bounding boxes. To remove redundancy and retain the optimum oriented bounding box that was closest to the ground-truth, non-maximum suppression (NMS) was performed. Through this method, we could obtain more robust and stable results in some challenging circumstances.

The overview of the vehicle detection framework is shown in Figure 2. The details of the mentioned parts will be introduced in the following sections.

### 3.2. Fast Oriented Region Search

In this section we propose a novel method based on a window scoring strategy and orientation estimation with a box refinement.

#### 3.2.1. Edge Boxes NG Features Re-Ranking

**Figure 3:**
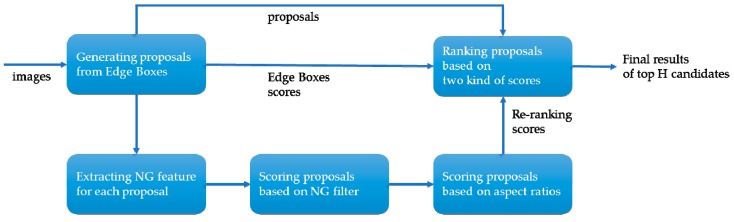
Workflow of EBNR.

First, we located the horizontal region proposals. It was obvious that using an original sliding window search was not advisable. Generally, a good objectness algorithm aims to generate proposals that could cover all of the objects present in the image. Simultaneously, the speed of the algorithm and the number of the proposals should be acceptable. In Hosang [23], several existing popular object proposal generating methods were collected and some experiments were done to evaluate the performance of them in different conditions. Three of them draw our attention owing to their speed and detection rate: Edge Boxes (EB) [24], Binarized Normed Gradients (BING) [25], and Selective Search (SS) [26]. However, none of them gets as good a recall as in the generic detection task (e.g., the Pascal Visual Object Classes (VOC) Challenge) due to the scale of target being extremely small and the high complexity of background in the aerial images. Therefore, inspired by the edge scoring strategy in EB and the normed gradients (NG) features in BING, we propose an effective and efficient method named Edge Boxes NG features re-ranking (EBNR), which integrates the advantages of EB and BING and achieves a better performance. The workflow is shown in Figure 3, which displays the processes of EBNR.

**Figure 4:**
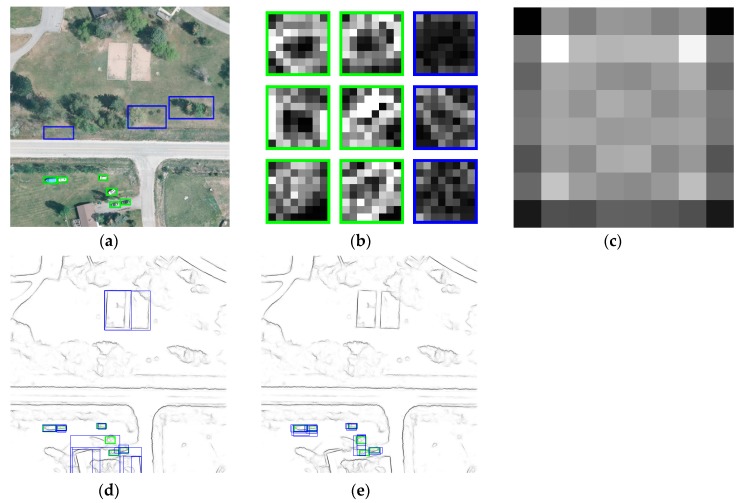
Instance specific proposals: (**a**) source image, (**b**) NG features, (**c**) learned model, (**d**) proposals using EB, and (**e**) proposals using our model.

EB and BING both belong to window-scoring-based methods according to J. Hosang. In Cheng [25], an observation was made that generic objects with a good enclosing boundary can be differentiated using NG features. They further designed a feature called BING, which requires only a few atomic operations and achieves an extremely fast speed. Whether in generic objects or vehicle detection, the recall of BING is very high at an intersection-over-union (IoU) threshold below 0.5, but it drops sharply with the IoU threshold increasing. We supposed that BING resizes the scales of images to achieve a sliding-window approach and this leads to imprecise localization. As for EB, it generates a pool of bounding boxes by sampling in a sliding window manner on the structured edge map, which achieves a good comprehensive performance. However, directly applying the output candidates of EB in the vehicle task has issues when the recall decreases significantly in a small number of proposals, e.g., 500, 1000, or 2000. As Figure 4d shows, the target of vehicle detection in UAV image is extremely small, where some proposals containing multiple targets are very likely to be produced. Without models to constrain the sizes of small targets, proposals with more edges when sliding the window will get higher scores, and it leads to an undesirable result. Here we argue that both the scoring strategy based on edge groups and the object feature properties should be considered. Unlike other features like Haar-like, SIFT, or HOG, BING is quite fast when extracting and training with SVM, and it is insensitive to changes of scale and aspect ratio.

As shown in Figure 3, given an input image, we first use EB to generate a pool of initial bounding boxes $\left\{B_{i}\right\}$ by ranking and retaining the top H boxes with the highest scores, represented by:

(1) $$B_{}^{EB}={\left\{B_{i}|E_{i}\right\}}_{H}$$

where $E_{i}$ is a normalized and scored number of edge groups wholly enclosed by the bounding box $B_{i}$. Then, each window is scored with a linear model $w\in {\mathbb{R}}^{64}$ (Figure 4c), which is obtained at the training stage:

(2) $$s_{l}=\langle w,g_{l}\rangle$$

(3) l=(x,y,w,h)

where $s_{l}$, $g_{l}$, and l(x,y,w,h) are filter score, NG feature, and the location (including the position and the size) of a bounding box, respectively. Some aspect ratios of width and height (e.g., 400,100) were much less likely than others (e.g., 100,80) to hold a single object instance, especially in vehicle detection where the target is usually fixed. Therefore, we divided the initial bounding boxes into groups according to their aspect ratios. The *r* is defined as:

(4) r=(w,h)

We set some common fixed values, e.g., 1/3, 1/2.5, 1/2, and the aspect ratio of a bounding box is assigned to the nearest value of *r*. The re-ranking score can finally be defined as:

(5) $$o_{l}^{re}=v_{r}\cdot s_{l}+t_{r}$$

where $v_{r},t_{r}\in \mathbb{R}$ are coefficient and bias terms learned separately from each fixed aspect ratio *r*. 

Notice that, not only does this produce a set of ranked scores after the re-ranking procedure, but also EB has a set of scores based on the edge map. Although simply using the re-ranking score lets the recall make an obvious improvement in the following experiments, we tried to use EB scores to constrain the re-ranking score to achieve better performance. When a bounding box has a high re-ranking score and a low Edge Box score, its final score should be significantly penalized. Taking this into consideration, we offer here two weight functions: linear and Gaussian forms, which makes the method become EBNR-L and ENBR-G, respectively.

The linear form is defined as:

(6) $$o^{final}=\alpha o^{re}+\beta o^{EB}$$

and Gaussian form is defined as:

(7) $$o^{final}=o^{re}\cdot e^{\frac{-{\left(o^{EB}-\mu \right)}^{2}}{2\sigma^{2}}}$$

where α, β, μ, and σ are parameters; and $o^{final}$, $o^{re}$, and $o^{EB}$, is the final score, re-ranking score, and EB score, respectively. In this paper, we set μ to 0.3, σ to 0.8, and α and β in a dynamic interval after we evaluated the impact of different parameters on the experimental results.

Finally, our approach can be described according to:

(8) $$B={\left\{B_{i}|o_{l}^{final}\right\}}_{H}$$

#### 3.2.2. Vehicle Orientation Estimation

Intuitively, vehicle targets are relatively fixed in shape, where most of them are similar to rectangles. Thus, the Radon transform [27] is introduced to estimate the local orientation in the image patches. Inspired by the Radon transform processing method in the texture analysis field, we used it here to determine the principal direction of a target from a proposed region. For a bounding box containing a single vehicle target that can be described as I(x,y) with linear structures E(x,y), the general Radon transform can be defined as:

(9) $$R(r,\theta )=\int_{D}E(x,y)\delta (r-xcos\theta -ysin\theta )dxdy$$

It can be considered that *R* is the result of several lines doing line integrals for *E*(*x*, *y*) in a plane. The line is defined as r=xcosθ+ysinθ, where r is the distance between a certain point in the structured edge map and the origin point geometrically. θ is the angle between the line r and the *x*-axis, which has a range of [0, 180). D denotes the whole image plane. δ is the Dirac delta function, which is zero all the time except at the origin:

(10) $$\delta (t)=\{\begin{array}{cc} +\infty & t=0\\ 0 & t\neq 0 \end{array}$$

**Figure 5:**
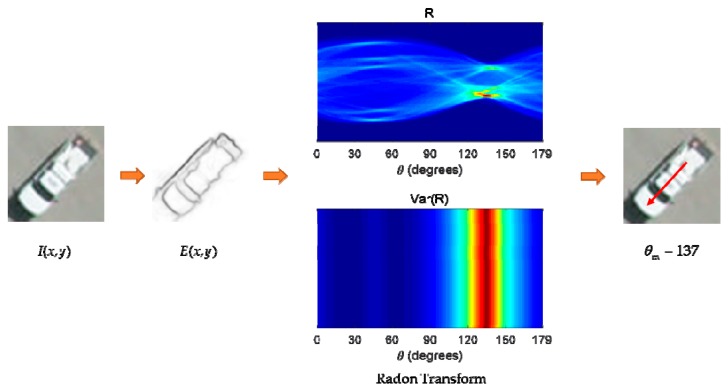
Vehicle orientation estimation.

The Radon transform reveals the component of lines at different angles. It is easy to draw the conclusion that the principal direction of a patch is the direction that has the most concentrated R component in the image, which can be obtained by calculating the maximum variance of R values. Let $\theta_{m}$ denote the principal direction. Then, we have:

(11) $$\theta_{m}=\max(\mathrm{Var}(R(r,\theta )))$$

where Var(R) is the variance of *R*. An example is shown in Figure 5, where *E*(*x*, *y*) is computed using a structured edge in EBNR phase, and R and Var(R) are shown by color maps where the red color represents the largest value. The red arrow indicates the estimated orientation of the horizontal proposal.

#### 3.2.3. Region Symmetrical Refinement

After we obtained the horizontal bounding boxes and their principal orientation, the next step was to find an inclined rectangle to enclose the target. Since the target may lie in any possible orientation (e.g., along the diagonal), simply rotating the horizontal bounding box aligning the target axis to the principal orientation using an anti-clockwise rotation of $\theta_{m}$ is not able to get an accurate result. Inspired by the horizontal box refinement algorithm proposed by Chen [28], we here present a two-step method to find the oriented bounding boxes that are along the principal orientations of objects and enclosing objects with the highest IoU overlaps.

**Figure 6:**
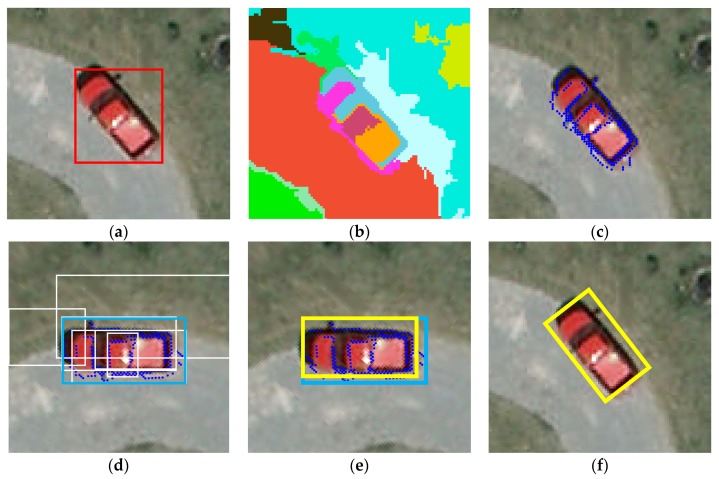
Oriented bounding box refinement: (**a**) horizontal bounding box, (**b**) superpixel segmentation, (**c**) Coarse Contours representation, (**d**) rotating points to get potential bounding box and superpixel set box, (**e**) box refinement in principal orientation, and (**f**) final result. Notice that, in (d) the rotation operation was not for the whole image but for the points only. In (d) and (e)**,** the enclosing bounding box of the coarse contours, superpixel set, and refined bounding box are shown in blue, white, and yellow, respectively.

Given a horizontal bounding box, the basic idea was to find potential oriented bounding boxes along their principal orientations, and then apply a box refinement that uses superpixels to adjust the boundaries of the box. As shown in Figure 6b, We first obtained the over-segmentation of the image, which consisted of superpixels $S_{}$ using the algroithm in Felzenszwalb [29]. More specifically, finding a potential oriented box that is close to ground truth will have a better impact in subsequent steps. Hence, the definition of a straddling degree (SD) in Chen [28] was utilized to quantize the proportion of the overlapping area as follows:

(12) $$\mathrm{SD}(s,b)=\frac{\left|s\cap b\right|}{\left|s\right|}$$

where the *s* is a subset of superpixels S, b is a bounding box, |s| is the area of a subset, and |s∩b| is the intersection area. By setting a threshold σ (usually 0.9 in practice), we can gather the coordinates of most of the superpixels in a horizontal bounding box, making it a new blob to represent the target inside the box:

(13) $$S_{\mathrm{bl}}=\left\{s\in S|\mathrm{SD}(s,b)>\sigma \right\}$$

Intuitively, the potential oriented box can be obtained by rotating the points in the blob to the basic axis and calculating the minimum enclosing box, and then rotating the enclosing box back to its principal orientation. To reduce the computational complexity, we here propose an alternative form of approximation, namely Coarse Contours, which is defined as:

(14) $$S_{c}=\left\{(p_{i,j_{1}},p_{i,j_{2}})\in S_{\mathrm{bl}}\right\}$$

where $p_{i,j_{1}}$ and $p_{i,j_{2}}$ are the two points with the smallest and largest abscissa of each row in the image. As shown in Figure 6c,d, this representation is sparse and concise, but enough to support us to get the enclosing bounding box of the blob in any orientation via more efficient computation than using the entire blob. By rotating the coarse contours of the blob and superpixel set to the principal orientation $\theta_{m}$, we can obtain a potential box and several superpixel boxes. As shown in Figure 6e,f, we only needed to rotate back the refined box in the horizontal orientation to its principal orientation and get the final result.

For a potential bounding box $b^{\circ }$, the goal of box refinement is to output a new box that has the highest overlap with the ground truth target. For this purpose, we can manipulate the boundaries of the potential bounding box based on its overlap with the superpixel boxes. To this end, we calculated the superpixels of inner set $S_{in}$ and straddling set $S_{st}$ of initial box $b^{\circ }$, which are defined as follows:

(15) $$S_{in}=\left\{s\in S|\mathrm{SD}(s,b^{\circ })=1\right\},S_{st}=\left\{s\in S|0<\mathrm{SD}(s,b^{\circ })<1\right\}$$

In other words, $S_{in}$ represents the superpixels entirely enclosed by $b^{\circ }$, and $S_{st}$ represents the superpixels both inside and outside $b^{\circ }$.

Then, the process involved merging superpixels greedily from the nearby straddling set. By sorting the subsets in the straddling set $S_{st}$ based on the IoU overlaps, its subsets $\left\{s_{1},\cdots s_{K}\right\}$ satisfied:

(16) $$O\left(b(S_{in}\cup \{s_{i}\},b^{\circ })\right)\geq O\left(b(S_{in}\cup \{s_{j}\},b^{\circ })\right),\forall \;i<j$$

where $b(S_{mg})$ denotes the minimum box enclosing the merged superpixels $S_{mg}$ and $O\left(b(S_{mg}),b_{o}\right)$ denotes the IoU overlap of two boxes. By expanding the boundaries of the box from $b(S_{in})$ to the closest one to the initial bounding box, we obtained a new bounding box $b^{⋆}$ that not only aligns with the superpixel boundaries, but also has the highest overlap with the given box $b^{\circ }$. 

Additionally, a multi-threshold superpixel merging algorithm was performed if the potential bounding box had a low IoU overlap with the object, where the box alignment process was unhelpful.

Given an aligned box *b*^⋆^ and a threshold *δ*, the straddling expansion is defined as follow:

(17) $$S_{\delta }(b^{⋆})=S_{in}(b^{⋆})\cup \{s\in S|\mathrm{SD}(s,b^{⋆})\geq \delta \}$$

By setting different threshold δ, we can obtain several different new bounding boxes $\hat{b}$, which is the minimum box enclosing the old boxes $S_{\delta }(b^{⋆})$. Since a fixed threshold may not be the best optional parameter for all aligned boxes, multiple thresholds $\delta_{i}$ are introduced to get a better performance, where $\delta_{i}$ is the set containing 0.1×i,i=1,2,⋯5, in practice. This generates five sets of bounding boxes as a consequence. Each set is sorted by adding a scoring principle where ${\hat{b}}_{i}$ is scored with value i×R, and R is a random number in (0,1]. In order to output the candidate boxes with highest overlaps and control the number of outputs, NMS is applied after sorting the bounding boxes.

Different from the way of representing a horizontal bounding box, the oriented bounding box could be denoted as follows:

(18) $$b=(x_{c},y_{c},w,h,\theta )$$

where $x_{c}$ and $y_{c}$ are the coordinate of center in the box, w and h are the width and height of the box, and θ is the angle between the principal orientation and coordinate axis. Since the width and height was invariant, and the angle was recorded as the principal orientation $\theta_{m}$, we only needed to calculate the center position of the box after rotating back.

**Figure 7:**
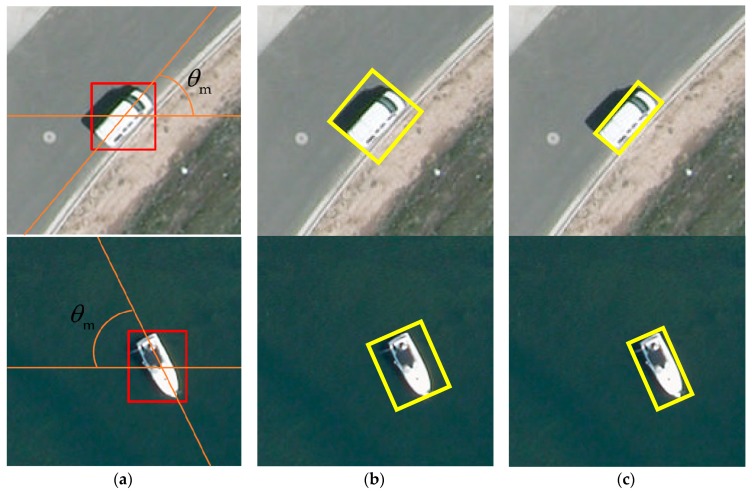
(**a**) Horizontal bounding box with principal orientation, (**b**) result of directly rotating, (**c**) and result of our method. Illustration of comparing directly rotating a horizontal bounding box and using our method.

The benefit of this box refinement method is that it only computes superpixels for one time and does not require calculating features (e.g., color, light intensity, and texture) to measure similarity between regions. As for the rotating operation, only a few points in Coarse Contours were rotated back. Therefore, this method computes efficiently with an extremely fast speed, and can get quite a high IoU overlap with ground-truth as the comparison, as shown in Figure 7. Notice that targets in aerial images usually have shadows. Obviously, results without shadows are more accurate and beneficial for the subsequent procedures. Generally, the pixels of the shadow are usually grouped into an independent superpixel set owing to the over-segmentation algorithm we used, which is based on the strategy of measuring the similarities between pixels, and our method can obtain good results in this case. In some particular cases, for instance, the color of the target is exactly the same as the color of the shadow, and so our method may get a result with shadows due to the pixels of the target and the shadow being grouped into a common superpixel set, which is relatively rare for the reason that targets and shadows are still different at the pixel level because of illumination, structure, and noise, etc. As for the small object around the target, for instance, a rod is around the stern of the ship in Figure 7, and the small size makes it very likely to be divided into the merged box $b^{⋆}$ during the superpixel merging process due to the sorting principal being based on a comparison of the IoU overlap after merging different superpixel subsets, which makes the results include the small object. Many small objects are originally part of the targets; if not, it will not have a great impact on subsequent processing since the classification model we designed is robust for such a small variation.

### 3.3. Feature Definition

**Figure 8:**
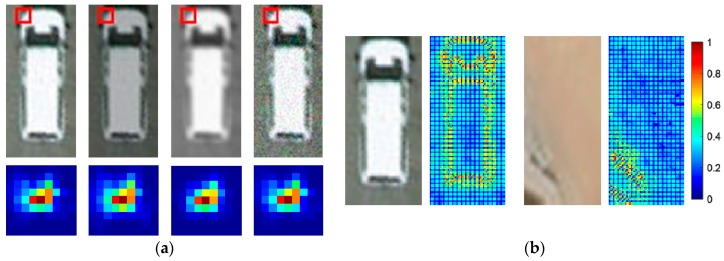
Illustration of LSK features. (**a**) LSK features of a *P* × *P* window (marked in the red boxes) in various circumstances. The vehicle image patches from left to right are source image, brightness change, blur change, and white Gaussian noise change, respectively. (**b**) Example of the difference of LSK features between a car and background shown by color maps.

In the literature, various features have been used to discriminate vehicles, most famous among them are Haar-like, SIFT, and HOG features. The LSK feature was originally proposed in kernel regression for image processing and reconstruction [30], mostly to perform denoising and deburring. Then, it was further adapted to generic object detection and has displayed good performance. These features have been compared by Seo [31], where the results indicated that the LSK feature has a more powerful capability to discriminate generic objects in experiments. In addition, the LSK feature is robust and stable as a descriptor, even if the image is in the presence of noise, blur, and brightness changes, as shown in Figure 8. Thus, with these benefits, the LSK feature is chosen to be extracted for the proposals to complete the image representation in this paper.

Essentially, the basic idea of the LSK feature is to compute the local similarity of a pixel to its neighboring pixels to obtain the local structure by quantizing the differences in pixel value level, and then construct the shape and size of a kernel with the structure information. The definition of a local kernel K is as follows:

(19) $$K(x)=\frac{G(H_{i}^{-1}(x_{i}-x))}{\det(H_{i})},i=1,\cdots ,P^{2}$$

where x and $x_{i}$ are the 2-D vectors of spatial coordinates and the coordinates of a pixel, $P^{2}$ is the number of pixels in a local window P×P, G(⋅) is the Gaussian function, and $H_{i}$ is the steering matrix, which is defined as:

(10) $$H_{i}=hC_{i}^{-\frac{1}{2}}$$

where h is a global smoothing parameter, and $C_{i}$ is a covariance matrix computed using gradient vectors in the local window. With the steering matrices and the Gaussian function, the expression of LSK leads to the following form:

(21) $$K(x)=\frac{\sqrt{\det(C_{i})}}{2\pi h^{2}}\exp(-\frac{{\left(x_{i}-x\right)}^{T}C_{i}(x_{i}-x)}{2h^{2}})$$

Given an image patch that is sampled with M×N bins, each bin has a kernel K calculated in the P×P window. As a consequence, we obtained a vector gathered with M×N kernels as the descriptor of the image patch. In order to maintain consistency, the kernel at the patch indexed by j is normalized according to:

(22) $$W^{j}(x)=\frac{K{\left(x\right)}^{j}}{\sum_{i=1}^{P^{2}}K{\left(x\right)}^{j}},\left\{ \begin{array}{l} j=1,\ldots ,MN\\ i=1,\ldots ,P^{2} \end{array}\right.$$

Intuitively, the kernel is relatively sparse and structured, where most powers of the descriptor are concentrated at a few clusters. To reduce dimensionality, PCA is introduced to reserve the characteristic of the structure. By reshaping, we can obtain $w^{j}$, which is the column-stacked version of $W^{j}$. Let W be the matrix consisting of vectors $w^{j}$, which are densely computed from the image:

(23) $$W=[w^{1},\ldots ,w^{MN}]\in {\mathbb{R}}^{P^{2}\times MN}$$

Then, the PCA is applied to W so that we can retain the largest d principal components that form the columns of a matrix $A\in {\mathbb{R}}^{P^{2}\times d}$. Finally, the lower dimensional feature F consisting of $f^{j}$ is computed, which is obtained by projecting W onto A:

(24) $$F=[f^{1},\ldots ,f^{MN}]=A^{T}W\in {\mathbb{R}}^{d\times MN}$$

### 3.4. VLAD Representation

The VLAD representation has the ability to enhance the expression of an image by aggregating descriptors into a fixed-size dimension based on a local principle in feature space. In Jegou [32], VLAD was estimated to have better performance than BoW and more efficient than the Fisher kernel, which makes it a proper way to represent LSK features in our work.

The idea of VLAD is quite like the idea of BoW, which involves the grouping of image descriptors into typical codewords, and then counting the frequency of word appearances in the image. A similar idea is used in vehicle detection in our work, where the LSK features of image patches are viewed as codewords. Then, different from BoW, VLAD accumulates residuals for each codeword, which means it measures the difference between the descriptor and the codeword. The implementation of the VLAD representation with LSK features is composed of the following steps.

**Figure 9:**
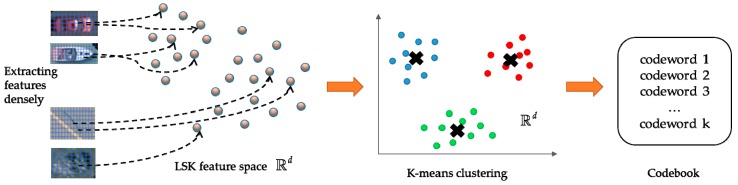
Illustration of obtaining the codebook in the VLAD representation.

Given a large amount of samples in the training database, to provide reasonable efficiency and accuracy, the LSK features are densely extracted on the image patches where the window size and sampling step are both set to five pixels. As shown in Figure 9, to construct a codebook consisting of codewords, the K-means algorithm is used to cluster *k* clusters of LSK features, which are regarded as codewords. Setting the capacity of the codebook with different *k* could have a different impact on the results (there is a discussion about this in Section 4.5.3), in this paper, the *k* is set to 300 based on practical experiments.

After the codebook $C=\left\{c_{1},\ldots ,c_{k}\right\}$ is learned, each local descriptor $x_{j}$ of $X=\left\{x_{1},\ldots ,x_{n}\right\}$, which is extracted from the image, is associated to its nearest codeword $c_{i,j}=\mathrm{NN}(x_{j})$. The VLAD representation is obtained by summing over all the local descriptors of the image:

(25) $$V=\sum_{i=1}^{k}\left(x_{j}-c_{i,j}\right)$$

where the $x_{j}$ is a component of the local descriptors and $c_{i,j}$ is the corresponding nearest codeword. Assuming the dimension of LSK feature is d, then the indices i=1,…k and j=1…d index the codeword and the LSK feature component, respectively.

Usually, the VLAD encoding is normalized before being used, and several normalization methods are introduced in the open-source library VLFeat. In this paper, we normalize the VLAD representation by taking the square root of the normalization, which means applying the function $\mathrm{sign}(z)\sqrt{\left|z\right|}$ to the matrix V.

### 3.5. Vehicle Object Category Classifiers

Selecting proposals to label as training samples is critical to the performance of the model. It is clear that oriented bounding boxes enclosing the ground-truth can be labeled as positive samples. Likewise, the proposals containing background that have no relevant objects should be negative samples. As for the proposal that partially overlaps a vehicle object, the usual practice is to set a threshold to regard those boxes below the threshold as negative samples. The selection of the threshold is very important. In this paper, we set the IoU overlap threshold to 0.2, which allowed us to get best result in practice.

**Figure 10:**
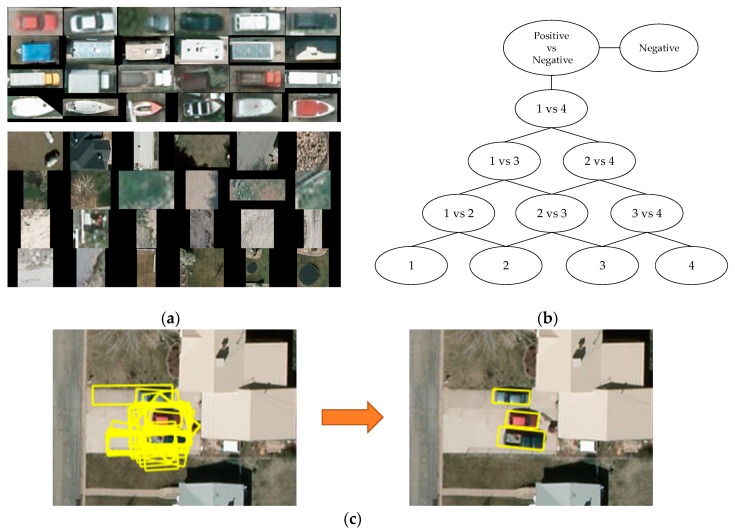
Classification for vehicle categories: (**a**) training samples of positive (up) and negative (bottom), (**b**) illustration of classifying four vehicle categories with DAG SVM, and (**c**) illustration of reserving detection results using NMS.

Once LSK features are represented by VLAD and training labels are applied, the SVM was used to complete the classification. In reality, SVM is designed to be a binary classifier training with positive and negative samples. Obviously, positive samples can be selected in ground-truth data as a certain amount. However, the selection of negative samples is controversial. In the literature, some researchers choose to let the background and the rest of ground-truth of other vehicle class be the negative samples for the SVM of this class. In this way, the number of negative samples is much larger than the number of positive samples, and we found this imbalanced distribution may cause unpredictable error and the performance of the classifier could be significantly reduced sometimes. Thus, we introduce a method based on DAG SVM to solve this problem. This method is composed of two steps. First, we trained an SVM classifier to distinguish proposals of the object and background, where the positive samples contained the whole ground-truth of all the classes, while the negative samples contained the same number of background proposals during the training phase. Second, a DAG SVM was established by training the classifier only once between different categories. An example of classifying four categories is shown in Figure 10. As illustrated, if the number of categories is n, we could easily find that the classifiers under the “Positive vs. Negative” chart in the DAG graph have the relation of the combination in mathematics, which can be calculated with the formula $\frac{n!}{m!(n-m)!}$. In this case, m=2, and along with adding the first classifier “Positive vs Negative”, the total number of the classifiers is $\frac{n(n-1)}{2}+1$. Meanwhile, there is no data imbalanced distribution issue since the numbers of different categories of positive samples and negative samples can be easily manipulated.

Additionally, different SVM kernel functions can have different effects on the results. In this paper, we select the chi-square kernel with the stochastic dual coordinate ascent solver because the performance of classifiers in DAG SVM can be improved more this way than generally using linear SVM models with the SGD solver.

After the classification, every proposal was classified into a specific category. As shown in Figure 10c, it was usually the case that many oriented bounding boxes partially contained the target. To eliminate redundancy, the NMS was performed to reserve the box having the highest overlap with the ground-truth. Soft-NMS [33] has made great progress in preventing missing mutually occluded targets. Our NMS algorithm was modified from Soft-NMS, which made it more effective at orienting bounding boxes. Different from a horizontal bounding box, orientation should be a factor to be considered in evaluating the score of the box for an oriented bounding box. Based on observation, oriented bounding boxes with isolated orientations that have a huge difference with the orientation of their nearby boxes could possibly appear as noise and they should be scored lowly.

## 4. Experiments and Discussions

In this section, we discuss experiments that were conducted to verify the performance of our vehicle detection method. First, we conducted a comparative experiment on the capability of EBNR and other state-of-art algorithm in generating proposals. Then, we ran the whole detection framework in two different datasets. Finally, we conducted experiments to evaluate our method. The algorithm was programmed using Matlab and C++ with open-source libraries: VL-FEAT and OPENCV. All the experiments were carried out on a computer equipped with Intel Xeon CPU E3-1230 v6 (4 cores, 3.5 GHz), 32 GB memory, and NVIDIA Quadro P2000.

### 4.1. Datasets

Two datasets were used in these experiments. Vehicle detection in aerial imagery (VEDAI) [34] is a publicly-available dataset captured over Salt Lake City, which comes in two image resolutions 512 × 512 and 1024 × 1024 in two image types: colored and infrared. Each image has 5.5 targets on average, which occupied about 0.7% of the total number of pixels in the image. A total of 1210 images include different backgrounds, i.e., grass, mountains, deserts, and rural and urban areas. They are divided into nine different categories, i.e., “boat,” “camping car,” “Car,” “others,” “pick-up,” “plane,” “Tractor,” “truck,” and “van.” Every target in VEDAI has been annotated manually in terms of $\left(x_{c},y_{c},w,h,\theta \right)$ in the same way we recorded the oriented bounding boxes mentioned in Section 3.2.3. Notice that the orientation interval of some categories was [−π,π], whereas the interval of the others was [0,π). For statistical convenience, we normalized the orientation interval to [0,π) for all the categories in VEDAI. In order to be able to perform repeatable validation, images among each subset were divided into 10 folds, where each fold had an individual training set and testing set. In all the experiments using VEDAI, the images were selected in color mode with a 1024 × 1024 size.

The other dataset we used was the Munich dataset [35], which was collected in the Munich city, Germany. It is composed of 20 large optical aerial images captured using a DLR 3K system, where half of the images are selected for training and the other for testing. The backgrounds are full of complexity, including but not limited to roads, buildings, playgrounds, and fields. Annotated vehicles are categorized into several types, for instance, car, truck, bus, etc. As for the same reason in VEDAI, we also normalized the orientation interval to [0,π) for all the categories. The time in computation cost was much longer for the Munich dataset than for the VEDAI dataset due to the image size (e.g., 5616 × 3744).

### 4.2. Metric

We analyzed the experimental results with two popular metrics: the precision-recall curve (PRC) and average precision (AP). To reveal the performance of the models using the two metrics, recall and precision are required, where the definition of them are as follows:

(26) $$\mathrm{Recall}=\frac{\mathrm{TP}}{\mathrm{TP}\;+\;\mathrm{FN}}=\frac{\mathrm{TP}}{\mathrm{GT}}$$

(27) $$\mathrm{Precision}=\frac{\mathrm{TP}}{\mathrm{TP}+\mathrm{FP}}$$

where TP, FP, FN, and GT indicate the true positives, false positives, false negatives, and ground-truth number of objects, respectively. Recall and precision reflect the correct probability and false alarm rate of the detection, respectively. In reality, AP is the accumulation of area under the PRC, which is a comprehensive metric for the model. Additionally, different from the definition in the Pascal VOC Image Detection Challenge and the Microsoft Common Objects in Context (MS COCO), a detection was considered correct only if its center lay in the ground-truth and its IoU overlap was higher than the threshold.

### 4.3. Proposal Generating Results

We compared the EBNR with three other popular proposal generating algorithms to illustrate its superiority. Following Hosang [23], we evaluated the performance of methods with the recall metric, which is computed as the ratio of the number of bounding boxes above a certain IoU overlap threshold to the number of ground-truth bounding boxes. The x-recall graph indicates the recall at IoU threshold x with a particular number of proposals (1000). The recall–proposals curve indicates the number of proposals to achieve the desired recall, where in this paper, the IoU threshold of recall was set to 0.7 for comprehensive performance considerations. Furthermore, in order to depict the performances of a method between IoU 0.5 to 1, a metric namely average recall (AR) [23] was introduced, which calculates recalls under different IoU thresholds and performs with an average value. To evaluate the accuracy of localization, we also computed the average best overlap (ABO) [26], which is computed as the average value of the highest IoUs that overlap with the ground-truth.

**Figure 11:**
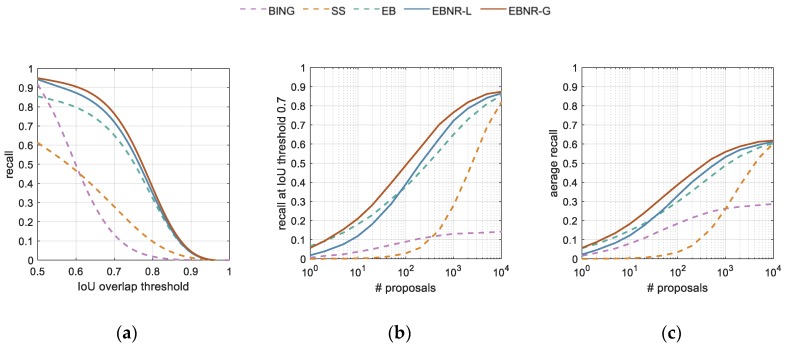
Recall on VEDAI dataset for three popular methods (in dashed lines) and two variations of our method (in solid lines): (**a**) 1000 proposals per image, (**b**) recall at 0.7 IoU, and (**c**) average recall. The method from top to bottom is shorthand for Binarized Normed Gradients, Selective Search, Edge Boxes, Edge Boxes NG features re-ranking. Best viewed in color.

The plots and statistics of recall are reported in Figure 11 and Table 1. The experiment is conducted on the VEDAI dataset repeatedly, and the final results are the mean value of the 10 folds. For the three existing methods, it is clear that EB performed better than the other two methods. We believe that this was mainly because the structured edge feature that is used by EB is more suitable in the situation of detecting small targets. Unlike detecting generic objects, the recalls obtained from Selective Search (SS) were unexpectedly low, where the strategies of SS used for measuring the similarity in different ways probably became ineffective for small vehicle detection. As for BING, similar to generic detection, the recalls were acceptable at low IoU, whereas they dropped dramatically as the IoU threshold increased. We suppose that BING implementing a sliding window by resizing the proposals could possibly be the cause of this result. These characteristics help to support the correctness of our choice of EB for improvement.

Our method EBNR was performed in two variants: EBNR-L and EBNR-G, which represent the linear or Gaussian function used in the re-ranking part, respectively. Visibly, EBNR-L already obtained a higher recall at IoU 0.7 than the other three methods when the number of proposals was larger than 100 in Figure 11b. Moreover, our advanced EBNR-G performed best in every evaluation, except it cost a bit more time than BING. It was only necessary to compute a Gaussian function during the ranking phase, so the time consumption was nearly the same as EBNR-L. Considering the huge improvements that were achieved, our EBNR-G was found to be the most appropriate algorithm in this paper.

### 4.4. Vehicle Detection Results

#### 4.4.1. VEDAI Results

As we mentioned above, the VEDAI dataset had 10 folds, so we generated detection results for all the categories (except for “plane” and “others” because of the lack of positive samples) and calculated the mean values of all the folds. A SVM + HOG detector and Deformable Parts Model (DPM) performed well between the methods used in Razakarivony [34] where the VEDAI dataset was built, and Faster Region-based Convolutional Network method (Faster R-CNN) [36] is a state-of-art deep learning detection approach.

Comparisons of AP for several methods are reported in Table 2, which shows that our method outperformed a classic hand-crafted feature-based detection method in all the categories. Even if compared with the deep learning approach [36], our method still had advantages in some categories, such as “camping car,” “pick-up,” and “truck.” It seems reasonable that categories like that with a strong structure are more likely to be recognized than categories like “boat” and “tractor,” which usually show some randomness or chaos in their structure. As for some categories, such as “boat,” the Faster R-CNN performed better than our method, and we supposed this was mainly because it contained multiple networks that could discover the underlying features, which also brought more complexity in computation.

#### 4.4.2. Munich 3K Results

**Figure 12:**
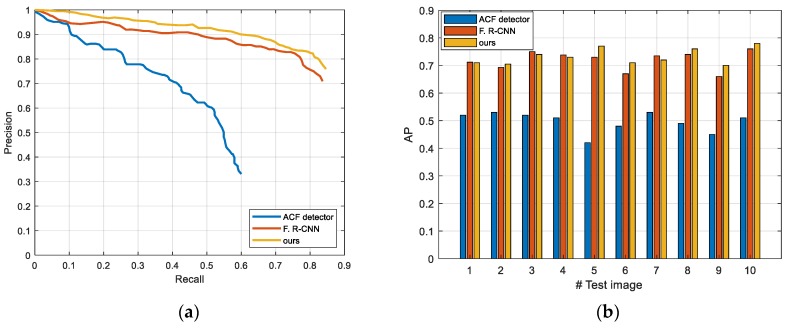
Results on the test-set of the Munich 3K dataset: (**a**) precision-recall curves, and (**b**) performance comparisons of 10 test images.

The test-set of Munich 3K only provides aerial images without annotations. To validate the correctness of the results, we annotated the ground-truth with oriented bounding boxes for the objects manually. To compare with other methods, we treated all the vehicle objects as the same category. Following Tang [37], we selected an ACF detector (used in K. Liu [38]), Faster R-CNN, and our method to conduct the experiments. Table 3 shows the numerical detection results of test images on Munich 3K, and Figure 12 shows the results of all the test images. Compared with the ACF detector and Faster R-CNN, our method achieved a better performance. Compared with Faster R-CNN, our method had more true positives, whereas there were negligibly more false positives, but the curve of our method in Figure 12a dropped more slowly when the recall grew, which also indicates that our method was superior to the other two methods.

### 4.5. Evaluations and Discussion

#### 4.5.1. Evaluation of Time Complexity

To evaluate the speed of our method, we conducted an experiment with a set of testing images to compare the computational time with the method in Moranduzzo [21] and the method in Ammour [39] during the testing stage. These images were the same images used in the two above methods, except we cropped the images to a 1024 × 1024 grid. We directly used the model trained on the Munich dataset so that we could validate the migration capability. As Table 4 shows, the results were similar to the experimental results we achieved above. For the evaluation of algorithm efficiency, we must mention that advanced GPU equipment could accelerate the calculation time dramatically. For our method, we used the GPU operation on the steps of feature extracting and VLAD representation, and achieved a speed of 10 fps. However, we only had a way to get the experimental results of the method in Moranduzzo [21] and the method in Ammour [39] under CPU versions. For fairness, we needed to ensure that all hardware conditions were the same, so we here list the computation time using a CPU. The result shows that our method was faster than the other two methods owing to needing less computation, and achieved a higher recall and nearly the same precision results.

#### 4.5.2. Evaluation of LSK Features

In this paper, the LSK features contains the following parameters: the window size *p* was set to 7 × 7, the sampling step was set to three pixels, and the Gaussian parameter *h* was set to 2. The number of principal components *d* was set to 4 during the PCA phase, as recommended in Seo [31].

To validate the function of the LSK feature, we compared the performance with SIFT and HOG, which are popular features widely used in detection tasks. At the feature extracting phase, we computed Dense-SIFT and HOG instead of LSK features, and then used the same strategies in all other phases to run the experiments on the collected dataset in Section 4.4.1. The result shows that AP decreased by 4 points if replaced with Dense-SIFT, and AP decreased by 5 points if replaced with HOG.

We suppose the reason was mainly because the mechanism of LSK made it more suitable to recognize the objects with compact structures, especially in the images disturbed by noise, blur, and illumination. However, in most experiments, LSK also has relatively precision since it produces more false positives, and is more likely to classify objects have similar shapes to cars mistakenly, e.g., a roof of a house with a small size.

#### 4.5.3. Evaluation of VLAD

Most classic methods directly extracting features without encoding representation, where some of them use a BoW to represent features. To examine the prior version of VLAD we used in this paper, we compared the performance differences. As Table 5 shows, both recall and precision were increased with encoding representation. Instead of directly using features to represent an image, image encoding representation finds a way to analyze the composition of the image. Therefore, it is more robust and invariant to changes, especially if the images contain a wide range of variations. VLAD and BoW have similarities in their basic idea since they both represent an image by quantizing the frequency and distance of features in the codebook. However, VLAD focuses on accumulating variables while BoW uses a histogram. In our vehicle detection approach, we could see that VLAD had more advantages than BoW from the results. Our method could classify an image patch more correctly.

**Figure 13:**
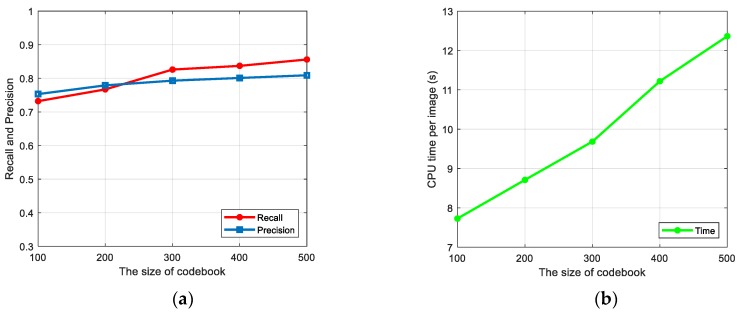
Results of evaluating VLAD with different sizes of codebook: (**a**) recall and precision with different sizes of codebook, and (**b**) time consumption with different sizes of codebook.

In the VLAD model, the size of codebook k is a significant parameter. To evaluate the influence of different k toward the final results, we conducted experiments with the same processes except setting different k for the codewords. As Figure 13a shows, we set k to be 100, 200, 300, 400, and 500, and display the corresponding recall and precision results with red and blue curves. Intuitively, the detection result grows as the size of codebook k increases. However, the cost of increasing k is that the computation becomes more complicated, which in turn makes the time consumption larger, as shown by the green curve in Figure 13b. From the trend of the red and blue curves, it is easy to observe that the improvement grew gently after 300. As for the green curve, the time had a positive correlation with the number of the codewords, especially as the slope of the curve became steeper after 300, which meant the time consumption increased rapidly when k was bigger than 300. Therefore, by considering a good trade-off between performance and efficiency, k was set to 300 in this paper.

#### 4.5.4. Discussion about the Quantitative Examples

**Figure 14:**
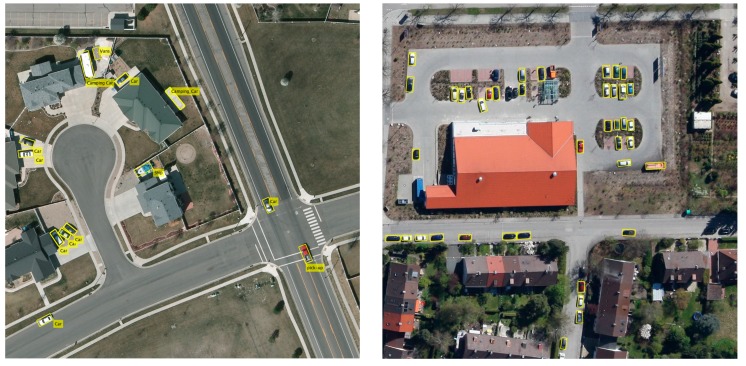
The quantitative detection examples of VEDAI (left) and Munich 3K (right) dataset.

By observing the example results shown in the left of Figure 14 for the VEDAI dataset, we found that our detection results were desirable, but some backgrounds were incorrectly detected as vehicles; for instance, a roof of a building was detected as a van. We think this was mainly due to the appearance of the roof being similar to the van in the overhead view in the VEDAI dataset, where even human beings could make a misjudgment in this situation. Though our method achieved a very good performance, it sometimes went wrong for this kind of hard example. As for the quantitative example shown in the right of Figure 14 for the Munich 3K dataset, there were some missing vehicles in the detection result. For instance, a black car failed to be detected. We think this was mainly because of the large size of the image in the test-set. Larger size means larger search area to generate proposals and more complicated backgrounds, which increases the difficulty of accurate detection. The dark color of a black car was very similar to the shadow, which could also be one of the reasons for the failure in the detection. To improve the robustness of the model and the results of the detection, it is worthwhile to try to increase the diversity of the training samples using some tricks in the existing dataset, or to modify the training methods (such as adding the hard example mining method).

## 5. Conclusions

In this paper, we have proposed a robust vehicle detection method for aerial images. The method was composed of four major procedures. First, we designed an algorithm to generate ORoIs called a Fast Oriented Region Search. By using EBNR, proposals presented as the horizontal bounding boxes were proposed accurately and quickly. The principal orientation of each horizontal box was estimated based on the Radon transform, and then by applying a Region Symmetrical Refinement, we obtained oriented bounding boxes enclosing the objects. The second procedure was extracting vehicle features. We opted to densely compute LSK features for the priority of robustness and invariance compared with classic image features such as SIFT and HOG. The third procedure was using the VLAD representation to aggregate the LSK features. It encoded a codebook of codewords from training samples that contained vehicles and backgrounds, and then accumulated the features of each image patch with the nearest codeword. VLAD quantizes the distances between the codewords and components of vehicles or backgrounds in the image patch, which is beneficial for the detection results, and experiments were conducted to validate this opinion. The final procedure was the classification: an offline training phase was performed to learn multiple classifiers of vehicle categories by using DAG SVM. During the detection phase, several oriented bounding boxes were generated from a test aerial image, then each patch was classified by applying the trained classier models. Finally, the NMS approach was performed to retain a single oriented bounding box for a vehicle object. We conducted experiments on two publicly available datasets: VEDAI and Munich 3K, and the results indicate the superiority of our method. We also applied evaluations for our method, the comparison results ascertain the significance of each procedure of the proposed method.

Although our vehicle detection method achieved a favorable performance for aerial images, there are still some aspects that can be improved. One is that the speed of our method could be accelerated by optimizing the algorithms in the framework. For instance, replacing the graph-based segmentation with other more efficient algorithms in EBNR. Furthermore, when detecting hard examples, such as vehicle targets partially occluded or having similar shapes to backgrounds, our method still has limitations in some circumstances. For the future work, we will be more concerned with the deep learning approaches and finding a way to further enhance the performance of our method.

## Figures and Tables

**Table 1:** Proposal results on VEDAI dataset. We evaluated 10 folds in VEDAI, where results are the mean values of 10 folds. The three metrics used were: average recall (AR), recall at IoU of 0.7, and average best overlap (ABO), which are recorded for three budgets of proposals: 500, 1000, and 2000. Values in bold indicate the best performances.

Method	# Proposals = 500			# Proposals = 1000			# Proposals = 2000			Time (sec)
	AR	70%-Recall	ABO	AR	70%-Recall	ABO	AR	70%-Recall	ABO	
BING	24.7	12.2	59.7	26.3	13.1	60.7	27.2	13.4	61.3	**0.07**
SS	15.7	15.0	43.4	26.1	27.9	56.6	38.4	44.8	66.6	19
EB	43.2	56.3	67.0	48.9	65.0	71.5	53.6	72.9	74.7	0.3
EBNR-L	48.1	62.8	72.5	53.3	72.2	75.3	57.0	78.6	77.0	0.36
EBNR-G	**52.1**	**70.2**	**73.9**	**55.9**	**76.6**	**76.0**	**58.8**	**81.9**	**77.4**	0.36

**Table 2:** Detection AP (%) on VEDAI (10 folds). The tags from left to right represent the categories of “boat,” “camping car,” “car,” “pick-up,” “tractor,” “truck,” and “van,” respectively. The best-achieving results are given in bold.

Method	Boa	Cam	Car	Pic	Tra	Tru	Van
SVM + HOG	32.2	33.4	55.4	48.6	7.4	32.5	40.6
DPM	26.1	41.9	60.5	52.3	33.8	34.3	36.3
F. R-CNN	**66.2**	72.7	77.7	74.8	**54.4**	66.7	69.9
Our Model	60.3	**74.5**	**79.7**	**77.6**	39.5	**69.7**	**72.4**

**Table 3:** Statistics of the detection results between different methods on the Munich 3K dataset.

Method	GT	TP	FP	Recall	Precision
ACF detector	5892	3078	2143	52.2%	58.9%
F. R-CNN	5892	4487	976	76.2%	82.1%
Our Model	5892	4719	1006	80.1%	82.4%

**Table 4:** Performance and time consumption of different methods.

Method	Recall	Precision	Time Per Image (CPU)	Programming Language
Moranduzzo [21]	65.8%	53.1%	44.8 s	Matlab
Ammour [39]	79.4%	80.8%	64.6 s	N/A
Our Method	82.6%	79.3%	10.3 s	C++ & Matlab

**Table 5:** Effect of the use of image encoding representation.

Method	Recall	Precision
LSK	71.7%	67.1%
LSK + BoW	77.4%	72.8%
LSK + VLAD	82.6%	79.3%
